# Complete Stress–Strain Curves of Self-Compacting Steel Fiber Reinforced Expanded-Shale Lightweight Concrete under Uniaxial Compression

**DOI:** 10.3390/ma12182979

**Published:** 2019-09-14

**Authors:** Mingshuang Zhao, Bingxin Zhang, Pengran Shang, Yan Fu, Xiaoyan Zhang, Shunbo Zhao

**Affiliations:** 1 International Joint Research Lab for Eco-building Materials and Engineering of Henan, North China University of Water Resources and Electric Power, Zhengzhou 450045, China; zxyanzi@ncwu.edu.cn; 2 Henan Provincial Collaborative Innovation Center for Water Resources High-efficient Utilization and Support Engineering, Zhengzhou 450046, China; 3 School of Civil Engineering and Communications, North China University of Water Resources and Electric Power, Zhengzhou 450045, China; Z201710313234@stu.ncwu.edu.cn (B.Z.); prshang@stu.ncwu.edu.cn (P.S.); yanfu@stu.ncwu.edu.cn (Y.F.)

**Keywords:** steel fiber reinforced expanded-shale lightweight concrete (SFRELC), self-compacting, volume fraction of steel fiber, uniaxial compressive stress–strain curve, compression toughness ratio, calculation model

## Abstract

To expand the structural application of steel fiber reinforced expanded-shale lightweight concrete (SFRELC), a self-compacting SFRELC with high-workability was developed based on previous research. As part of the investigation, the present study focuses on the adaptability of formulas used for the complete stress–strain curves of steel fiber reinforced lightweight-aggregate concrete and conventional concrete under uniaxial compression. On the basis of mix proportion of SFRELC, self-compacting SFRELC was designed with the volume fraction of steel fiber as 0%, 0.4%, 0.8%, 1.2%, 1.6%, and 2.0%. Eighteen cylindrical specimens with dimensions of Φ150 mm × 300 mm were tested to measure the uniaxial compressive stress–strain curves of self-compacting SFRELC. Results indicated that, with the increasing volume fraction of steel fiber, the compressive strain at the peak-stress of the stress–strain curve increased, while the slope of the descending portion decreased. This increased the energy absorption of self-compacting SFRELC with a higher compression toughness. With a comparison of test results between four groups of calculation models, a group of formulas is selected to express the complete stress–strain curves of self-compacting SFRELC under uniaxial compression.

## 1. Introduction

In view of the brittleness of lightweight-aggregate concrete [1,2,3], and the utilization of local sintered expanded-shale as fine and coarse aggregates, a new concrete material called steel fiber reinforced expanded-shale lightweight concrete (SFRELC) was developed. To investigate SFRELC with different flowabilities, classified as plastic, flowing, and high flowing with the slump varied from 60 mm to 200 mm, a series of experimental investigations have been performed to study the mechanical properties, including compressive strength and toughness, tensile strength, flexural strength and toughness, deformation and modulus of elasticity, strength developments and complete stress-strain curves [4,5,6,7,8,9,10,11,12], carbonization and freeze–thaw resistance [10,13], as well as autogenous and drying shrinkage [14]. This confirmed the good mechanical performance, durability, and steady volume of SFRELC. To further enlarge the application of SFRELC, self-compacting technology is worthy of study, in order to support the mechanical vibration procedure during construction [15,16,17,18,19,20,21]. As a core point, the stress–strain relationship of self-compacting SFRELC is of great importance for the design of concrete structures. 

Based on previous studies, the stress–strain relationship of vibro-compacting lightweight aggregate concrete (LAC), with or without steel fiber, has been conducted by some researchers [11,22,23,24,25,26,27,28,29,30,31]. For LAC, with the peak stress varied from 18 MPa to 89 MPa, peak strains also presented an increasing tendency from about 2000 με to 6000 με, while the ratios of peak stress to strain, namely the secant modulus, significantly increased from 7 GPa to 15 GPa. The secant modulus was lower than 12–25 GPa of normal weight concrete (NWC), and this is due to the shape of the stress–strain curves being more linear as the peak stress of LAC increased with the higher deformability [22,23,24,25,26,27]. While in Campione’s study [25], the residual stress in the descending portion was about 26% of the peak stress for LAC and 67% for NWC at the same strain of 6000 με. Compared with the shape of the compressive stress–strain curves for LAC, more brittle behavior, namely a steeper and shorter descending curve, was observed with respect to NWC of the same strength.

Steel fiber was added to enhance the toughness of LAC, while the stress–strain relationship of steel fiber reinforced lightweight-aggregate concrete (SFRLAC) was significantly influenced. According to researches [11,25,29,30,31], hooked-ends steel fiber with aspect ratios of 60–67, or thin-plate cutting corrugated steel fiber with an aspect ratio of 27, was used to enhance the LAC with expanded-shale or other kinds of lightweight aggregates. The results showed that the shape of ascending curves was almost similar with the increased peak stresses at about 0–29%, the increased peak strains at about 15–55%, while the secant modulus presented a decrement of about 5–33%. Thus, the slope of the ascending portion of stress–strain in curves increased with the volume fraction of steel fiber, which indicates that the addition of steel fiber could promote the stiffness of SFRLAC. For the descending portion, slopes became smaller with the increasing volume fraction of steel fiber. The residual stresses of stress–strain curves for SFRLAC at a certain strain of 6000 με were about 47–76% of their peak stresses with the volume fraction of steel fiber of 2.0% [11,25,29,30]. The effect of steel fiber on the promotion of the toughness and ductility of SFRLAC connected with the type and aspect ratio of steel fibers. 

According to the experimental study of compressive stress–strain curves of vibro-compacting SFRELC, and the analysis a large number of test curves of LAC and SFRLAC, a unified stress–strain model was proposed for the prediction of complete compressive stress–strain curves of LAC and SFRLAC [11]. The proposed model was also modified and adopted by Ding et al. to get a good prediction for the complete compressive stress–strain curves of self-compacting steel fiber reinforced concrete (SFRC) under uniaxial compression [32]. Meanwhile, Aslani’s model [33,34] was proposed to predict the stress–strain relationship of self-compacting fiber reinforced concrete with different strengths, at different curing ages, in their own experiments. Cunha’s model [35,36] was put forward based on the calculation model in CEB-FIP model code 1990 [37] for self-compacting fiber reinforced concrete. The results showed that Cunha’s model had a good prediction for the ascending portion but underestimated for the descending portion of the compressive stress–strain curves in both Aslani’s and Ding’s researches. 

Based on the above reviews, no study was carried out on the compressive stress–strain model of self-compacting SFRELC, and the adaptability and applicability of the present models of LAC and SFRLAC have not been confirmed. Therefore, this paper focuses on the assessment and numerical prediction of the complete stress–strain curves of self-compacting SFRELC under uniaxial compression. Eighteen cylindrical specimens were prepared for self-compacting SFRELC with a varying volume fraction of steel fiber from 0% to 2.0%. Based on the test results, the proposed model, Aslani’s model, Cunha’s model, and the FIP model were verified to predict the stress–strain curve of self-compacting SFRELC. Meanwhile, the energy absorption ability of self-compacting SFRELC, as characterized by the compression toughness ratio, is also discussed. 

## 2. Preparation of Experimental Study

### 2.1. Raw Materials

**Figure 1:**
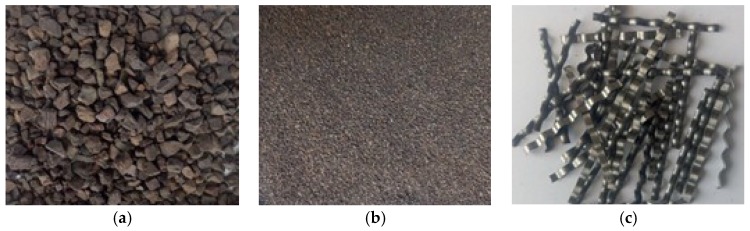
Figures of raw materials: (**a**) coarse expanded-shale; (**b**) ceramsite sand; (**c**) steel fiber.

**Figure 2:**
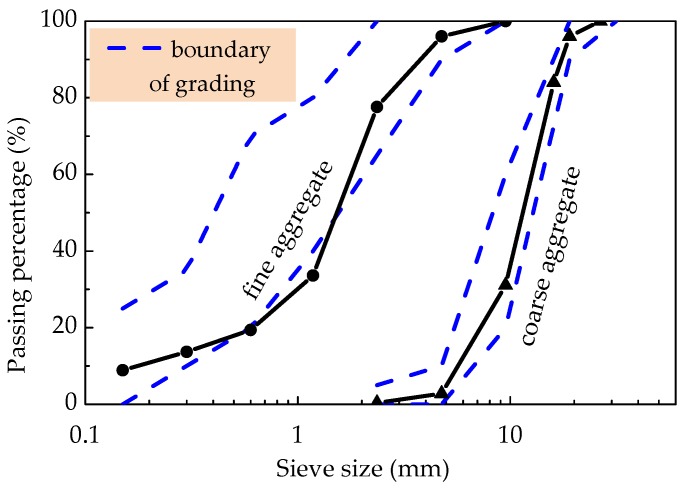
Grading curves of coarse expanded-shale and ceramsite sand.

**Figure 3:**
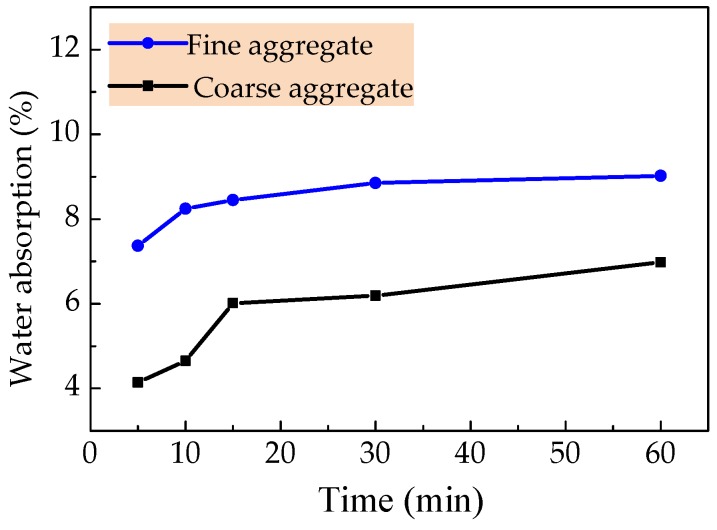
Water absorption curves of ceramsite sand and coarse expanded-shale.

Common Portland cement meeting the requirements of China code GB 175 was used [38], of which the physical and mechanical properties are presented in Table 1. Fly ash satisfying the indices of class-II specified in China code GB/T1596 [39] was used as the admixture, the density was 2070 kg/m^3^, the residual amount on a square hole sieve of 45 μm was 20%, and the water demand ratio was about 92%. As in the previous study [9,11] and presented in Figure 1a,b, high-strength sintering expanded shales in continuous gradation of 5–20 mm was used for the coarse aggregate, and the ceramsite sand, the byproduct of sintering expanded shale sieved as continuous gradation within 0.16–5 mm was used for the fine aggregate. The grading curves of coarse expanded-shales and ceramsite sand are drawn in Figure 2, their particle gradation basically met the requirement in China code GB/T 17431.2 [40]. The physical and mechanical properties are listed in Table 2, and the water absorption curves are displayed in Figure 3 to be used as the basis of selecting the presoaking time. At the same time, the 24 h water absorptions of expanded shale and ceramsite sand were measured as 7.45% and 9.75%. Due to the water absorptions within 1 h reaching 93.5% and 92.6% of 24 h water absorptions for expanded shale and ceramsite sand respectively, the rational presoaking time was chosen as 1 h. The steel fiber exhibited in Figure 1c was crimped cut with thin-plate, the length *l*_f_ = 36.7 mm, the equivalent diameter *d*_f_ = 1.35 mm, and the aspect ratio *l*_f_/*d*_f_ = 27.2. Others included the polycarboxylic acid superplasticizer with a water-reducing rate no less than 30%, and tap water as mixing water.

### 2.2. Mix Proportions

The water/binder ratio (*w*/*b*) was chosen as 0.30 to make the self-compacting SFRELC. The volume fraction of steel fiber *v*_f_ = 0%, 0.4%, 0.8%, 1.2%, 1.6%, and 2.0%, respectively. The volume ratio of ceramsite sand was 50% when *v*_f_ = 0%, 0.4%, and 0.8%, and then increased 0.2% with the increment of *v*_f_ = 0.4%. About 30% mass of cement was replaced by fly-ash. The details of mix proportion are presented in Table 3. Where the dosage of super-plasticizer was determined through testing according to the workability of fresh self-compacting SFRELC. As the saturated dry surface condition of expanded shale and ceramsite sand was adopted for the preparation of a fresh mixture of SFRELC, the dosage of presoaking water with 1 h water absorption was added additionally.

### 2.3. Test Methods

The expanded shale and ceramsite sand were presoaked for 1 h, and then mixed with cement, fly ash and steel fiber using a horizontal spindle forced mixer. The workability of fresh self-compacting SFRELC was measured according to the specifications in China code JGJ/T283 [41]. Three cubic specimens with dimension of 150 mm were used to test the compressive strength, three cylinder specimens with dimensions of Φ150 mm × 300 mm were used for measuring the uniaxial compressive stress–strain curves, and the mean values were adopted in accordance with the test method in China standards GB/T50081 [42], CECS13:2009 [43], and ACI 544.4R [44]. Specimens were cured in a standard curing room at (20 ± 2) °C temperature and 95% RH for 28 days before testing. 

**Figure 4:**
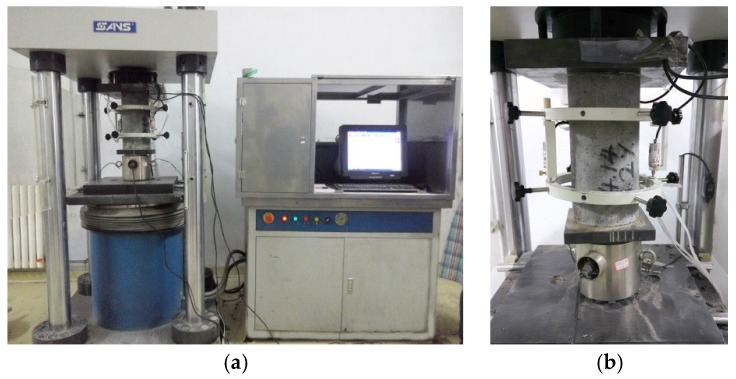
Equipment and instrument used in the test: (**a**) overall perspective; (**b**) testing device for load and deformation.

With the requirement of the sufficient rigidity of test machine to get a complete stress-strain curve of concrete under uniaxial compression [11], a 3000 kN electro-hydraulic servo universal testing machine with sufficient rigidity and higher loading control precision made by SANS Co. Ltd., as exhibited in Figure 4, was used as a loading device in this study. The loading speed was maintained as the strain rate among (20–50) × 10^−6^/s. The stress and strain data were measured by a load transducer and two displacement meters on two symmetric sides of each specimen.

## 3. Results and Analysis

### 3.1. Workability and Density

**Figure 5:**
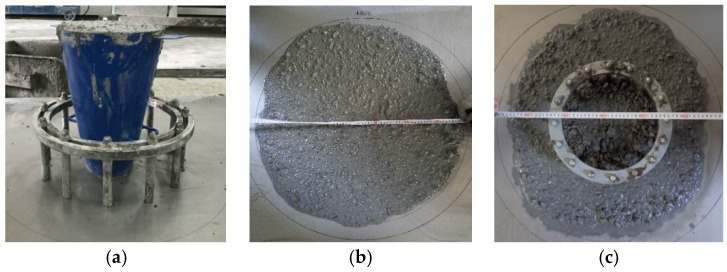
Tests for the workability of fresh self-compacting SFRELC: (**a**) testing device; (**b**) slump flow; (**c**) J-ring slump flow.

**Figure 6:**
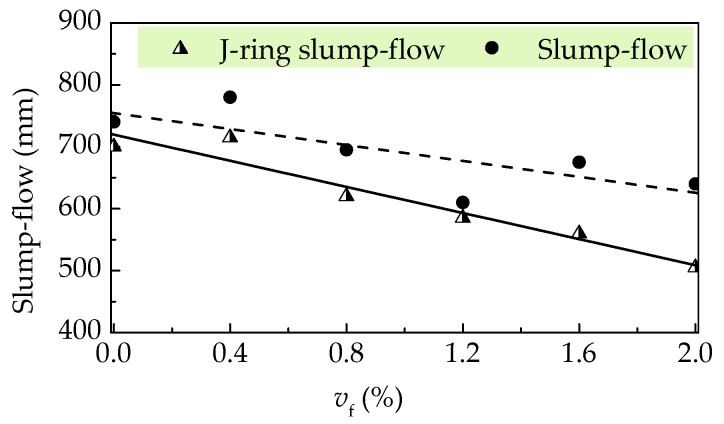
Slump flow with a varying *v*_f_.

**Figure 7:**
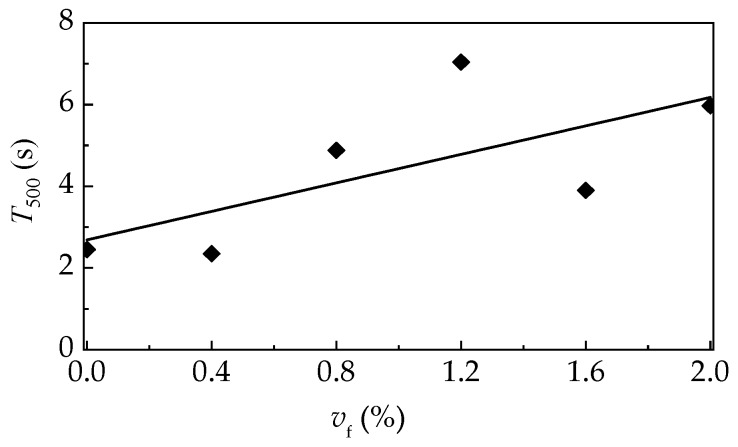
Slump flow time with a varying *v*_f_.

Figure 5 displays the inverted slump cone method for slump flow and J-ring slump flow tests to evaluate the filling and passing abilities of self-compacting SFRELC. Test results are illustrated in Figure 6 and Figure 7. Represented by the decreased slump-flow, J-ring slump flow, and the increased slump-flow time (*T*_500_), the filling ability decreases with the increase of *v*_f_. With the *v*_f_ increasing from 0% to 2.0%, the slump flow and J-ring slump flow deceased 13.5% and 27.9%, respectively, while the *T*_500_ increased from 2.35 s to 7.04 s. The maximum value of *T*_500_ took place corresponding to the minimum slump flow at *v*_f_ = 1.2%. This is due to the insufficient dosage of water reducer. The J-ring slump-flow expressed a faster reduction than the slump-flow, which indicated a fast declination of passing ability of self-compacting SFRELC with the increase of *v*_f_. In general, the slump-flows for all mixes of self-compacting SFRELC were 610–780 mm, which met the performance level *SF*1 and *SF*2 specified in China code JGJ/T283 [41]. J-ring slump flows were 505–715 mm, and the *T*_500_ was longer than 2 s. This met the requirements of a reinforced concrete structure cast by self-compacting SFRELC [34,36].

**Figure 8:**
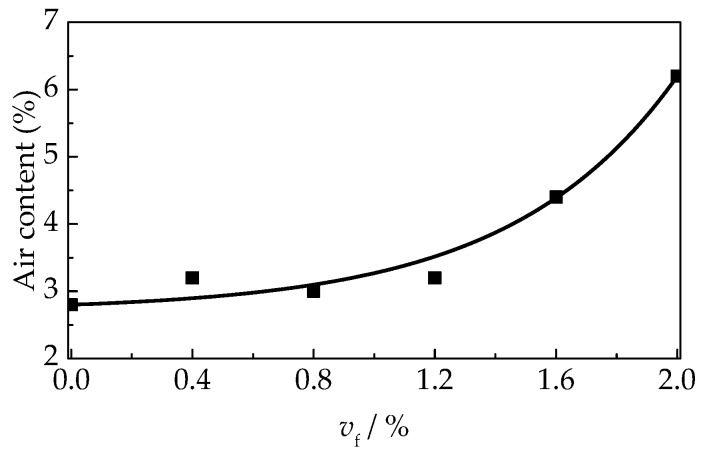
Variation of the air content with *v*_f_.

**Figure 9:**
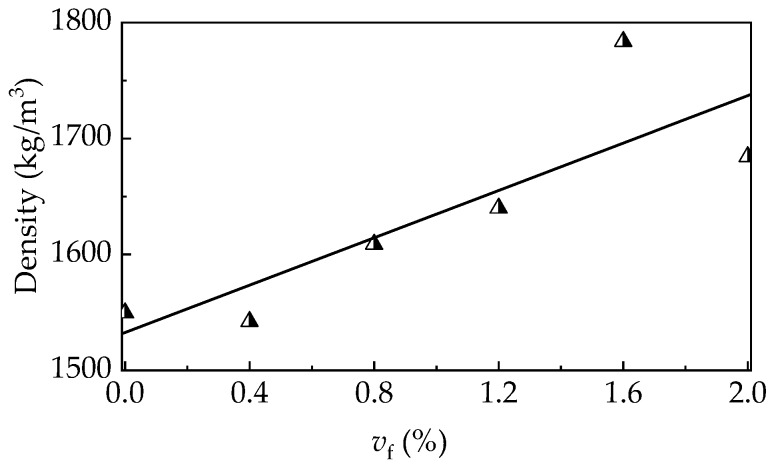
Variation of the density with *v*_f_.

As presented in Figure 8, the air content of self-compacting SFRELC were more and more sensitive with the addition of steel fiber, especially when *v*_f_ was over 1.2%. The air content grew slightly when *v*_f_ = 0–1.2% but had a great increase from 4.4% to 6.2% when *v*_f_ varied from 1.6% to 2.0%. However, the densities of self-compacting SFRELC increased with *v*_f_ despite the increase of air content. This is due to the larger density of steel fiber. It should be noted that the higher air content may lead the reduce of the densities of self-compacting SFRELC, as displayed in Figure 9. The dry density of self-compacting SFRELC ranges from 1542 kg/m^3^ to 1784 kg/m^3^. It is about 25.7–35.8% lower than that of NWC. 

### 3.2. Uniaxial Compressive Stress-Strain Curves of Self-Compacting SFRELC

**Figure 10:**
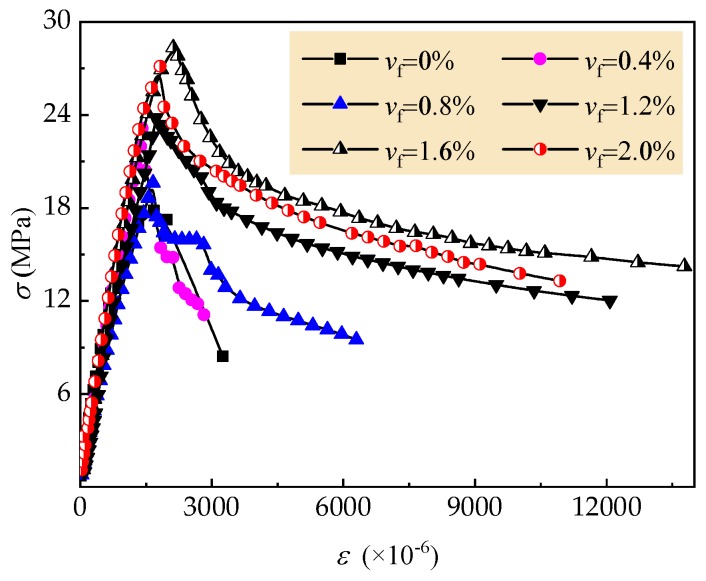
Stress-strain curves of tested self-compacting SFRELC with different *v*_f_.

Figure 10 presents the uniaxial compressive stress–strain curves of tested self-compacting SFRELC. With the increase of *v*_f_, the curves had a trend with a steeper slope at the ascending portion and slower slope at the descending portion. The peak-stress *f*_c,r_ and corresponding strain *ε*_c,r_ trend increased linearly. 

**Figure 11:**
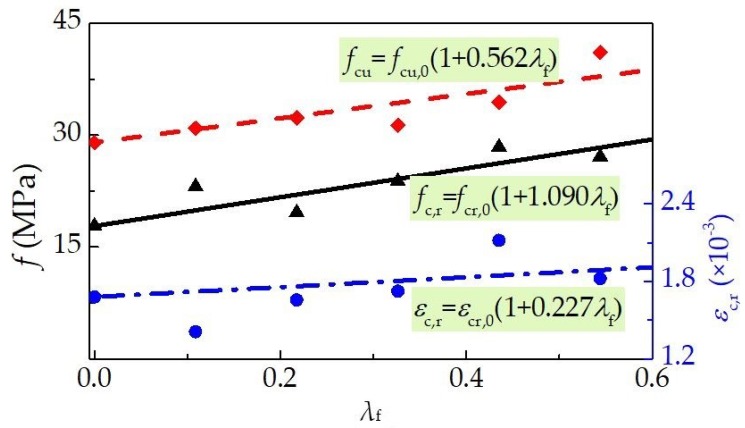
Variation of *f*_cu_, *f*_c,r_ and *ε*_c,r_ with a varying *λ*_f_.

The test results of peak-stress (*f*_c,r_) and peak strain (*ε*_c,r_) are summarized in Table 4 and exhibited in Figure 11, while the variation of cubic compressive strength *f*_cu_ is also displayed, in which the fiber factor *λ*_f_ = *l*_f_/*d*_f_·*v*_f_ = 27.2*v*_f_. It can be seen that the compressive strengths and peak strains increased with the *v*_f_ due to the enhanced restraining effect of steel fiber on the transversal deformation of the specimen. With the *v*_f_ varying from 0% to 2.0%, the increments of *f*_cu_, *f*_c,r_ and *ε*_c,r_ are 35.5%, 51.3% and 27.1%. According to the fitting formulas in Figure 11, the increment ratio of *f*_cu_, *f*_c,r_ and *ε*_c,r_ with the increase of *v*_f_ are 0.562, 1.090 and 0.227, respectively. This indicates that the effect of steel fiber on *f*_c,r_ is higher than that on the *f*_cu_, and the values of *f*_c,r_/*ε*_c,r_ namely the secant modulus increase with the addition of steel fiber. The slope of ascending portion increased with the increasing volume fraction of steel fiber. Values of *f*_c,r_ are about 0.6–0.8 times of *f*_cu_ in Table 4, which are lower than that reported in the experiments of Li et al. [7] of flowing SFRELC with the same raw materials and mix proportions. This may be attributed to the slower loading rate having a significant effect on the testing results. 

**Figure 12:**
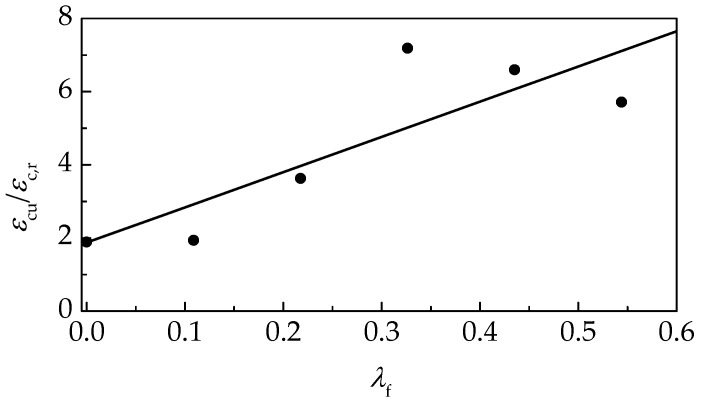
Change of *ε*_cu_/*ε*_c,r_ with *λ*_f_.

According to China code GB50010 [45], *ε*_cu_ was defined as the strain corresponding to 0.5*f*_c,r_ at the descending portion of stress-strain curves. The test results are presented in Table 4, and exhibited in Figure 12 as the relative values of *ε*_cu_/*ε*_c,r_ with a varying *λ*_f_. The obvious increasing relationship between *ε*_cu_/*ε*_c,r_ and *λ*_f_ indicates that the ductility of self-compacting SFRELC after breaking was promoted by the addition of steel fibers. With the *v*_f_ increased from 0% to 2.0%, *ε*_cu_/*ε*_c,r_ was from 1.87 to 7.11, 2.8 times increase. The maximum value was achieved at *v*_f_ = 1.2%. 

### 3.3. Compression Toughness

**Figure 13:**
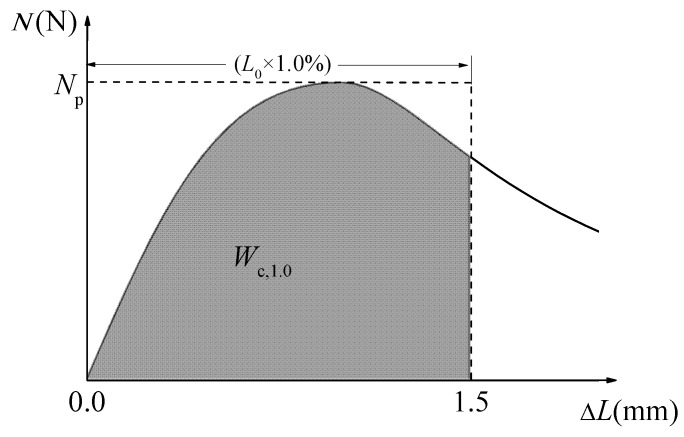
Calculation diagram for *R*_e,1.0_.

Table 4 summarizes the test results of compression absorbed energy (*W*_c,1.0_) and compression toughness ratio (*R*_e,1.0_). The *W*_c,1.0_ was calculated by the area under the compressive load-deformation curve within the uniaxial compressive deformation of 1.0% standard gauge length of 150 mm, as presented in Figure 13. *R*_e,1.0_ was used to evaluate the energy absorption ability of self-compacting SFRELC during compression deformation, which can be calculated as follows [9].

(1) $$R_{e,1.0}=\frac{W_{c,1.0}}{N_{p}\cdot L_{0}\times 1.0\%}$$

where, *N*_p_ is the peak compressive load, *L*_0_ is the standard gauge length.

**Figure 14:**
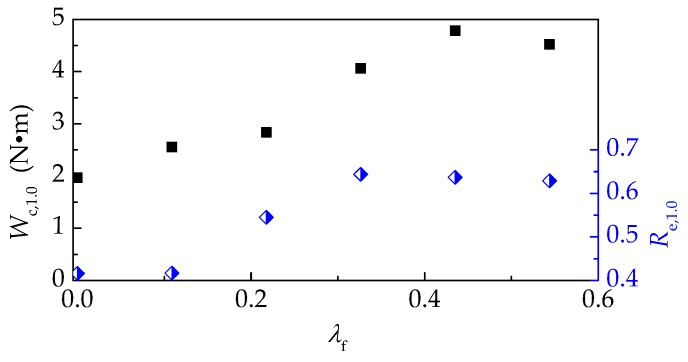
Variation of *W*_c,1.0_ and *R*_e,1.0_ with a varying *λ*_f_.

Figure 14 presents the variations of compression absorbed energy (*W*_c,1.0_) and compression toughness ratio (*R*_e,1.0_). With the *v*_f_ = 0.4%, the presence of steel fiber improves *W*_c,1.0_ about 30% but almost no enhancement on *R*_e,1.0_. This indicates that the crack-bridging effect of smaller amount steel fibers mainly reflects at the pre-peak to arrest the transversal expanded deformation. With the *v*_f_ varying from 0.4% to 1.2%, the toughening effect of steel fiber was outstanding, and the increments of *W*_c,1.0_ and *R*_e,1.0_ were 59% and 54.5%, respectively. When the *v*_f_ increased to 1.6% or 2.0%, *W*_c,1.0_ and *R*_e,1.0_ had no significant promotion. This may be due to the unfavorable effect of large content steel fibers on the compactness of self-compacting SFRELC, which results in the decrease of loading capacities after peak loads. According to previous test results [9], the *R*_e,1.0_ of vibro-compacting SFRELC increased, even when *v*_f_ was up to 2.0%. It can be said that the self-compacting SFRELC has a higher sensibility for steel fibers than the vibro-compacting SFRELC, and the volume fraction of steel fiber in self-compacting SFRELC should not be greater than 1.2%. 

## 4. Evaluation for Compressive Stress-Strain Curve of Self-Compacting SFRELC

### 4.1. The Proposal Model

According to the China code GB50010 and previous report [11,45], the complete stress-strain curve of lightweight aggregate concrete and vibro-compacting SFRELC can be expressed as Formulas (2)–(4). The ascending portion of the curves were calculated by Formula (2), and the coefficient *n* was determined by Formula (3). The descending portions of curves were fitted using Formula (4), in which *α*_c_ and *b* are statistical parameters relating to the shape of descending portion of the stress-strain curve of lightweight aggregate concrete. According to the calculation model in report [11], *α*_c_ is calculated by Formula (5). For vibro-compacting SFRELC, *α*_cf_ and *b*_f_ have the same meanings with *α*_c_ and *b*, and can be calculated by Formulas (6) and (7). 

When 0 ≤ $\frac{\varepsilon_{c}}{\varepsilon_{c,r}}$ ≤ 1,

(2) $$\frac{\sigma_{c}}{f_{c,r}}=\frac{n\frac{\varepsilon_{c}}{\varepsilon_{c,r}}}{n-1+{\left(\frac{\varepsilon_{c}}{\varepsilon_{c,r}}\right)}^{n}}$$

(3) $$n=\frac{E_{c}\varepsilon_{c,r}}{E_{c}\varepsilon_{c,r}-f_{c,r}}$$

When $\frac{\varepsilon_{c}}{\varepsilon_{c,r}}$ > 1,

(4) $$\frac{\sigma_{c}}{f_{c,r}}=\frac{\frac{\varepsilon_{c}}{\varepsilon_{c,r}}}{\alpha_{c}{\left(\frac{\varepsilon_{c}}{\varepsilon_{c,r}}-1\right)}^{b}+\frac{\varepsilon_{c}}{\varepsilon_{c,r}}}$$

(5) $$\alpha_{c}=0.00022f_{c,r}{}^{2.75}+0.746$$

(6) $$\alpha_{c,f}/\alpha_{c}=1/(1+2.43\lambda_{f})$$

(7) $$b_{f}/b=1/(1+0.386\lambda_{f})$$

where, *σ*_c_ and *ε*_c_ are the stress and the strain at any point of stress-strain curve, *n* is a material parameter that depends on the shape of stress-strain curves, *f*_c,r_ and *ε*_c,r_ are the peak-stress and the peak-strain respectively, *E*_c_ is the modulus of elasticity of concrete. *λ*_f_ is the fiber factor.

**Figure 15:**
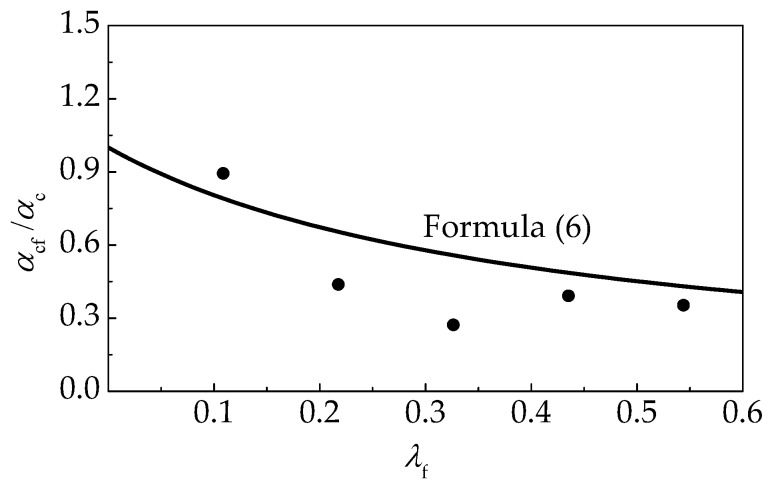
Relationship between *λ*_f_ and *α*_cf_/*α*_c_.

**Figure 16:**
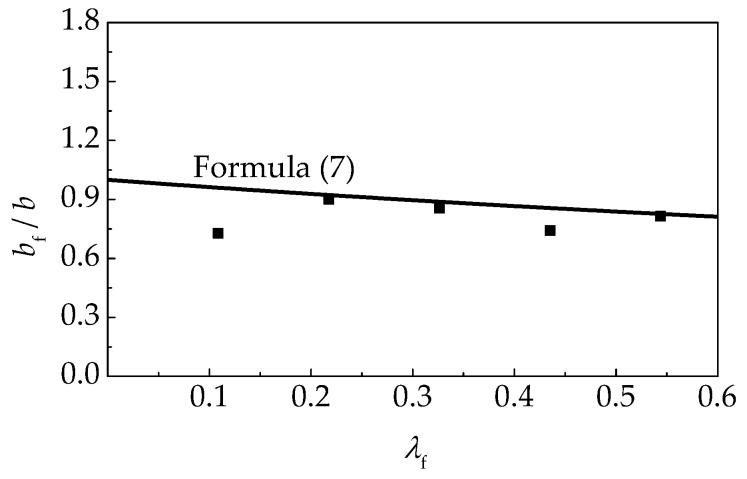
Relationship between *λ*_f_ and *b*_f_/*b*.

The tested values of *α*_cf_/*α*_c_ and *b*_f_/*b* for self-compacting SFRELC are displayed in Figure 15 and Figure 16. A good fitness can be achieved compared with the calculation results of Formulas (6) and (7). 

**Figure 17:**
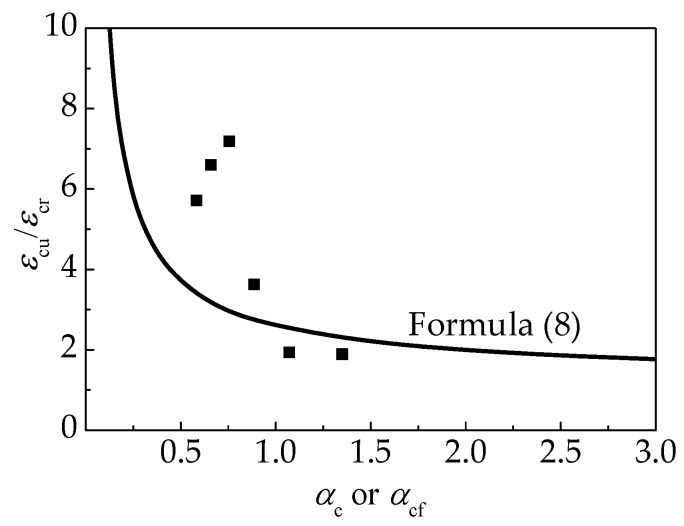
Relationship between *ε*_cu_/*ε*_c,r_ and *α*_c_/*α*_c,f_.

The relationship between *ε*_cu_/*ε*_c,r_ and *α*_c_ or *α*_c,f_ is shown in Figure 17, which fits the Formula (8) specified in China Code GB50010 for conventional concrete as follows [45],

(8) $$\frac{\varepsilon_{\mathrm{cu}}}{\varepsilon_{c,r}}=\frac{1}{2\alpha_{\mathrm{cf}}}(1+2\alpha_{\mathrm{cf}}+\sqrt{1+4\alpha_{\mathrm{cf}}})$$

### 4.2. Aslani’ Model

Aslani’s model for compressive stress–strain curves is the same in form with Formula (2) [32,33,34]. The material parameter *n* can be calculated by Formulas (9)–(14).

When 0 ≤ $\frac{\varepsilon_{c}}{\varepsilon_{c,r}}$ ≤ 1,

(9) $$n={\left[1.02-1.17(E_{c1}/E_{c})\right]}^{-0.74}.$$

When $\frac{\varepsilon_{c}}{\varepsilon_{c,r}}$ > 1,

(10) $$n={\left[1.02-1.17(E_{c1}/E_{c})\right]}^{-0.74}+(\rho +28\omega ).$$

where,

(11) $$\rho ={\left(135.16-0.1744f_{c,r}\right)}^{-0.46}$$

(12) $$\omega =0.83\exp(-911/f_{c,r})$$

(13) $$E_{c1}=\frac{E_{c}(\nu -1)}{\nu }$$

(14) $$\nu =\frac{f_{c,r}}{17}+0.8.$$

In these formulas, *E*_c1_ is the secant modulus, and *ρ*,*ω* are the coefficients corresponding to the *f*_c,r_ in Formula (10).

### 4.3. Cunha’ and FIP Model

The basic form for Cunha’s model and the FIP model [35,36,37] is presented as Formulas (15)–(17).

When $\varepsilon_{c}\leq \varepsilon_{c,\lim}$,

(15) $$\frac{\sigma_{c}}{f_{c}}=\frac{\frac{E_{c}}{E_{c1}}\frac{\varepsilon_{c}}{\varepsilon_{c,r}}-{\left(\frac{\varepsilon_{c}}{\varepsilon_{c,r}}\right)}^{2}}{1+(\frac{E_{c}}{E_{c1}}-2)\frac{\varepsilon_{c}}{\varepsilon_{c,r}}}.$$

When $\varepsilon_{c}\geq \varepsilon_{c,\lim}$,

(16) $$\frac{\sigma_{c}}{f_{c}}={\left\{\left[\frac{1}{\varepsilon_{c,\lim}/\varepsilon_{c,r}}\xi (\frac{1}{2\alpha }{)}^{2}-\frac{1}{{\left(\varepsilon_{c,\lim}/\varepsilon_{c,r}\right)}^{2}}\frac{1}{\alpha }\right](\frac{\varepsilon_{c}}{\varepsilon_{c,r}}{)}^{2}+\left[\frac{1}{\varepsilon_{c,\lim}/\varepsilon_{c,r}}\frac{2}{\alpha }-\xi (\frac{1}{2\alpha }{)}^{2}\right]\frac{\varepsilon_{c}}{\varepsilon_{c,r}}\right\}}^{-1}$$

(17) $$\xi =\frac{4\left[(\frac{\varepsilon_{c,\lim}}{\varepsilon_{c,r}}{)}^{2}(\frac{E_{c}}{E_{c1}}-2)+2\frac{\varepsilon_{c,\lim}}{\varepsilon_{c,r}}-\frac{E_{c}}{E_{c1}}\right]}{{\left[\frac{\varepsilon_{c,\lim}}{\varepsilon_{c,r}}(\frac{E_{c}}{E_{c1}}-2)+1\right]}^{2}}.$$

where, *ε*_c,lim_ is the limited strain at stress of *αf*_c,r_ which may be calculated by Formula (18).

(18) $$\varepsilon_{c,\lim}=\frac{1}{2}\left[(1-\alpha )\frac{E_{c}}{E_{c1}}+2\alpha \right]+{\left[\frac{1}{4}{\left[(1-\alpha )\frac{E_{c}}{E_{c1}}+2\alpha \right]}^{2}-\alpha \right]}^{0.5}$$

For Cunha’s model, the parameter *α* is related to the curing age *t* of concrete as presented in Formula (19), and value of *α* at curing age *t* = 28d is 0.9.

(19) $$\alpha =0.9\exp\left\{0.005\left[1-{\left(\frac{28}{t}\right)}^{1.16}\right]\right\}$$

For the FIP model, *α* = 0.5.

### 4.4. Fitness with Tested Curves

**Figure 18:**
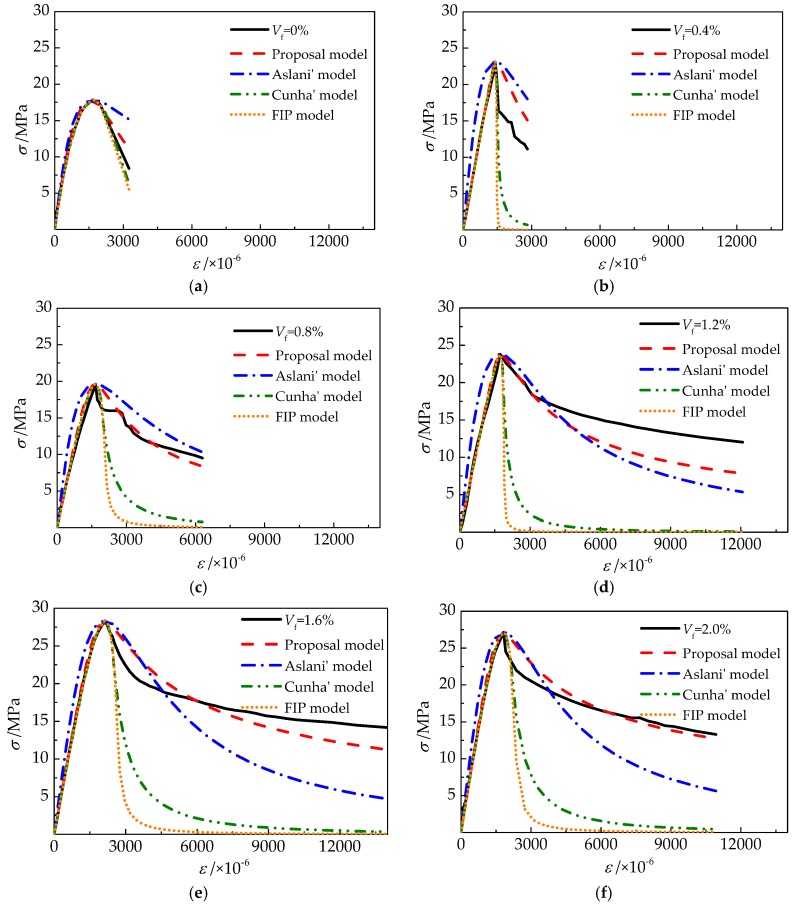
Test and calculated stress-strain curves of self-compacting SFRELC. (**a**) *v*_f_ = 0%; (**b**) *v*_f_ = 0.4%; (**c**) *v*_f_ = 0.8%; (**d**) *v*_f_ = 1.2%; (**e**) *v*_f_ = 1.6%; (**f**) *v*_f_ = 2.0%.

The tested compressive stress–strain curves are compared with the proposed model, Aslani’s model, Cunha’s model, and the FIP model, respectively. As exhibited in Figure 18, the good fitness by the four calculation models are in the order of the proposed model, Aslani’s model, Cunha’s model, and the FIP model. Based on the numerical analysis of test data, a similar conclusion will be obtained.

In this paper, the average predictive ratio of calculate to test stress (*AVG*) and coefficient of variation (*COV*) at the same strain are used to evaluate the degree of fitting. Formulas (20)–(21) are used to calculate the *AVG* and *COV* of fitting curves with the four models and results are displayed in Table 5.

(20) $$AVG=(\sum_{i=1}^{n}\frac{\sigma_{\mathrm{cal}}}{\sigma_{c}})/n$$

(21) COV=SD/AVG

where, *σ*_c_ is the stress at any point of stress-strain curve,; *σ*_cal_ is the calculate stress at the same strain with *σ*_c_, and *SD* is the standard deviation of *σ*_cal_/*σ*_c_.

For the proposed model, *AVG* and *COV* in the ascending portion are 0.935–1.101 and 0.019–0.185, while those in the descending portion are 0.991–1.303 and 0.065–0.156, with the volume fraction of steel fiber varying from 0% to 2.0%. For Aslani’s model, *AVG* and *COV* in the ascending portion are 0.875–1.665 and 0.090–0.273, and those in the descending portion are 0.811–1.413 and 0.051–0.356. For Cunha’s model, *AVG* and *COV* in the ascending portion are 0.875–1.098 and 0.023-0.181, and those in the descending portion are 0.189–0.947 and 0.138–1.557. For the FIP model, *AVG* and *COV* in the ascending portion are 0.458–1.098 and 0.023–0.345, and those in the descending portion are 0.092–0.300 and 0.281–2.765. These results display that nearly all of the four models but Aslani’s model have a good prediction for the ascending portion of the test curves. The proposed model is an effective predictor for complete test curves of self-compacting SFRELC. Aslani’s model shows a good fitness for curves of *v*_f_ = 0–0.8%, but slightly worse than the proposed model when *v*_f_ = 1.2–2.0%. Both Cunha’s model and the FIP model are inappropriate for the prediction of the descending portion in compressive stress–strain curves of self-compacting SFRELC.

## 5. Conclusions

Based on the test results and analysis, the following conclusions can be drawn:

(1) Self-compacting SFRELC with a slump flow of larger than 600 mm was prepared in this experiment. Slump-flow and J-ring slump-flow decreased while slump-flow time (*T*_500_) increased with the increase of *v*_f_. Drying densities increased about 12.2% with the *v*_f_ increased from 0% to 2.0%.
(2) With the increasing *v*_f_, the uniaxial compressive stress-strain curves of self-compacting SFRELC trends to be steep at ascending portion and a slower slope at descending portion. With the *v*_f_ varying from 0% to 2.0%, the increments of *f*_cu_, *f*_c,r_ and *ε*_c,r_ are 35.5%, 51.3% and 27.1% respectively. Values of *f*_c,r_ are about 0.6–0.8 times *f*_cu_, which may be attributed to the loading rate being slower than that used in the test of axial compressive strength. The residual strengths increased with the increase of volume fraction of steel fiber.
(3) With the *v*_f_ varied from 0.4% to 1.2%, the toughening effect of steel fiber was outstanding, and the increments of *W*_c,1.0_ and *R*_e,1.0_ are 59% and 54.5%, respectively. When the *v*_f_ reached to 1.6% and 2.0%, the *W*_c,1.0_ and *R*_e,1.0_ had no significant promotion. Therefore, the optimal *v*_f_ for self-compacting SFRELC can be taken as 1.2%.
(4) Based on the values of *AVG* and *COV* for the predictive ratios, and a comparison of calculations and test curves, the proposed model has a good fitness with the tested curves of self-compacting SFRELC, and Aslani’s model is slightly worse. Cunha’s model and the FIP model are suitable for the ascending portion, but inappropriate for the descending portion. As such, the proposed model is suggested in this paper.

## Figures and Tables

**Table 1:** Physical and mechanical properties of cement.

Density (kg/m^3^)	Water Requirement of Normal Consistency (%)	Setting Time (min)		Compressive Strength (MPa)		Flexural Strength (MPa)	
		Initial	Final	3d	28d	3d	28d
3085	26.4	160	245	29.4	54.7	6.2	9.4

**Table 2:** Physical and mechanical properties of expanded shale and ceramsite sand.

Particle Size (mm)	Apparent Density (kg/m^3^)	Bulk Density (kg⋅m^3^)	1 h Water Absorption (%)	Mud Content (%)	Cylinder Compressive Strength (MPa)
5–20	1262	827	6.98	0.7	7.4
0.16–5	1350	850	9.02	0.11	-

**Table 3:** Mix proportion of self-compacting SFRELC.

*w*/*b*	*v*_f_ (%)	Volume Ratio of Sand (%)	Cement (kg/m^3^)	Fly Ash (kg/m^3^)	Coarse Aggregate (kg/m^3^)	Fine Aggregate (kg/m^3^)	Super-Plasticizer (kg/m^3^)
0.30	0	50	408	175	424	417	0.210
	0.4	50			419	422	0.210
	0.8	50			413	428	0.210
	1.2	52			391	450	0.214
	1.6	54			368	472	0.222
	2.0	56			346	494	0.231

**Table 4:** Test result of compressive strength and toughness.

*w*/*c*	*v*_f_ (%)	*λ*_f_	*f*_cu_ (MPa)	*f*_c,r_ (MPa)	*ε*_c,r_ (×10^−3^)	*ε*_c,u_ (×10^−3^)	*E*_c_ (GPa)	*W*_c,1.0_ (N·mm)	*R*_e,1.0_
0.30	0	0	29.01	17.84	1.6801	3.1765	21.57	196766.1	0.42
	0.4	0.1088	30.92	23.13	1.4116	2.7317	17.14	255427.2	0.42
	0.8	0.2176	32.28	19.62	1.6569	6.0114	15.35	283445.1	0.55
	1.2	0.3264	31.30	23.8	1.7252	12.3964	15.13	406151.2	0.64
	1.6	0.4352	34.41	28.35	2.1190	13.9847	17.42	478505.5	0.64
	2.0	0.544	41.12	27.13	1.8224	10.4132	19.72	452164.4	0.63

**Table 5:** Calculation results of *AVG* and *COV*.

*v*_f_/%	Proposal Model				Aslani’s Model				Cunha’s Model				FIP Model			
	Ascending Portion		Ascending Portion		Ascending Portion		Descending Portion		Ascending Portion		Descending Portion		Ascending Portion		Descending Portion	
	*AVG*	*COV*	*AVG*	*COV*	*AVG*	*COV*	*AVG*	*COV*	*AVG*	*COV*	*AVG*	*COV*	*AVG*	*COV*	*AVG*	*COV*
0	1.017	0.177	1.08	0.144	0.875	0.198	1.234	0.309	0.875	0.181	0.947	0.138	0.458	0.345	0.807	0.281
0.4	1.018	0.023	1.303	0.101	1.603	0.2	1.413	0.143	1.019	0.023	0.359	0.981	1.019	0.023	0.153	2.152
0.8	0.988	0.134	1.025	0.088	1.38	0.125	1.158	0.051	0.969	0.122	0.456	0.837	0.969	0.122	0.3	1.396
1.2	1.101	0.127	0.867	0.156	1.665	0.273	0.832	0.265	1.098	0.13	0.189	1.557	1.098	0.13	0.092	2.765
1.6	1.018	0.019	0.991	0.111	1.266	0.09	0.811	0.356	0.995	0.023	0.318	1.091	0.995	0.023	0.198	1.739
2.0	0.935	0.185	1.036	0.065	1.153	0.168	0.825	0.315	0.911	0.177	0.304	1.099	0.911	0.177	0.182	1.685
